# Increasing the Strength and Impact Toughness of Carbon Steel Using a Nanosized Eutectoid Resulting from Time-Controlled Quenching

**DOI:** 10.3390/ma17153696

**Published:** 2024-07-26

**Authors:** Michail Brykov, Dariusz Mierzwiński, Vasily Efremenko, Vasyl’ Girzhon, Vadim Shalomeev, Oleksandr V. Shyrokov, Ivan Petryshynets, Olexandr Klymov, Oleksii Kapustyan

**Affiliations:** 1 Faculty of Engineering and Physics, National University Zaporizhzhia Polytechnic, 69063 Zaporizhzhia, Ukraine; vgirzhon@zp.edu.ua (V.G.); shalomeev@zp.edu.ua (V.S.); klymovo@zp.edu.ua (O.K.); aek@zp.edu.ua (O.K.); 2 Faculty of Materials Engineering and Physics, Tadeusz Kosciuszko Cracow University of Technology, Al. Jana Pawła II 37, 31-864 Cracow, Poland; 3 Physics Department, Pryazovskyi State Technical University, 49044 Dnipro, Ukraine; vgefremenko@gmail.com; 4 Division of Metallic Systems, Institute of Materials Research, Slovak Academy of Sciences, Watsonova 47, 04001 Košice, Slovakia; ipetryshynets@saske.sk; 5 Frantsevich Institute for Problems of Materials Science, National Academy of Sciences of Ukraine, Omeliana Pritsaka Str. 3, 03142 Kyiv, Ukraine; shyrokovav@gmail.com

**Keywords:** carbon tool steel, heat treatment, gradient nanostructure, low-carbon martensite, cementite, interlamellar spacing, strength, impact toughness

## Abstract

High-carbon steels are normally used as tool materials. The use of such steels for construction is limited due to their increased brittleness and poor weldability. However, it appears that high-carbon steels possess certain hidden reserves for enhanced plasticity and strength if properly heat-treated. An unconventional heat treatment was applied to carbon eutectoid steel (0.8 wt.% C) in order to increase its strength and impact toughness simultaneously. Samples for tensile and impact testing were held at 800 °C for different time ranges from 3 min to 9 min with subsequent cooling in oil. It was established that for each type of sample, an optimal holding time exists that is responsible for increased strength and high impact toughness. The hardness and microhardness levels of the surface and under-surface regions of the samples reached 390 HV after optimal heat treatment. An X-ray revealed a shift of the (211)α-peak to the lower 2-theta angles after heat treatment with the optimal holding time; this indicates an increase in carbon content in alpha solid solutions of approximately 0.12 wt.%. Thus, a nanostructured mixture of low-carbon martensite and thin cementite plates is formed in the under-surface region of carbon eutectoid steel after heat treatment, with a controlled holding time at the austenitizing temperature.

## 1. Introduction

Steels with high structural strength are essential materials for various aspects of human life. In emergency situations, human survival depends to a large extent on the properties of steels [1,2,3].

The strength of steels can vary widely depending on their chemical composition and processing methods. Thus, an important component of the structural strength of steels, the yield strength, can vary from 100 MPa (soft unalloyed low-carbon steels) to 1700 MPa, and even higher (high-strength steels of various alloying levels that have been subjected to heat treatment under special conditions) [4].

Traditional ways of strengthening steels include grain refinement [5], solid solution strengthening [6], strengthening with finely dispersed particles [5,7,8], and strengthening by plastic deformation (work hardening or strain hardening) [9]. These methods can increase the yield strength of steels to approximately 700 MPa [4]. Another strengthening mechanism is transformation strengthening, with results depending on the character of the final microstructure as well as the Peierls–Nabarro stress. 

According to the generally accepted designation [10], coarse-grained, ultrafine-grained, and nanocrystalline structures have component size ranges of 1–100 µm, 100–1000 nm, and 10–100 nm, respectively. A promising strengthening method, which in principle allows the combination of high strength and increased or high plasticity, is the refinement of grains to the level of one micrometer or even less [11]. An extremely valuable set of properties can be obtained in steels with structural components refined to the nanoscale [12,13].

An object is considered nanoscale if at least one of its linear dimensions is less than 100 nm [10]. A material is nanostructured if it contains layers less than 100 nm thick or particles less than 100 nm in size [4,14]. Nanostructured and ultrafine-grained steels have increased strength compared to those with relatively large grains [11,13,15,16,17].

Fine-dispersed structures and nanostructures in steels can be achieved by methods of severe plastic deformation [12,18], thermomechanical treatment [10,19,20,21,22,23,24,25,26], heat treatment [20], microalloying [5,7], and combinations of these methods. Severe plastic deformation is able to significantly refine the structural components of crystalline materials, particularly steels, and enhance YTS to a level of 2700 MPa [27]. The disadvantage of this method is technological complexity, while it is advantageous for applications to objects of relatively small dimensions.

Thermomechanical treatment combines plastic deformation and heat treatment. Unlike the case of severe plastic deformation, the degree of plastic deformation of the material during thermomechanical treatment is smaller, but the method allows the process parameters to be extensively changed and controlled, and it is actually used at most metallurgical enterprises.

Heat treatment is a very effective method for refining the structural components of steels. Since the heat treatment of steels mostly involves phase transformations of austenite into certain decomposition products (ferrite–cementite mixtures, martensite bainite), refining the austenite grain before the transformation naturally leads to a reduction in the size of the transformation products. Therefore, any treatment is useful that contributes to the refinement of the original austenite grain. One such method is repeated-phase recrystallization [28]. Controlled austenitizing (control of heating temperature, holding time, or both) is another effective method for refining the microstructure constituents of steels [29].

Microalloying with elements such as Nb, Ta, and V helps with the refinement of the microstructure of steels due to the formation of finely dispersed carbides and nitrides of these elements [8]. 

The application of the above-mentioned methods of microstructure refinement in steels in combination with well-known methods of strengthening (hardening to martensite/bainite) has led to the creation of a wide range of advanced high-strength steels. In the modern literature, high-strength steels are classified by generation [4].

First-generation advanced high-strength steels are low-alloyed [30]. Their structure combines ferrite, bainite, martensite, and austenite. Among the most prominent first-generation steels are two-phase ferrite–martensitic and ferrite–bainite steels, steels with a complex microstructure, and martensitic steels [30,31,32]. The maximum strength level of high-strength first-generation steels is 1500 MPa (up to 2200 MPa for martensitic steels), and the relative elongation ranges are between 5% and 30% [4]. The main problem with high-strength first-generation steels is a decrease in plasticity with an increase in strength. However, the refinement of ferritic and/or martensitic constituents can simultaneously increase strength and plasticity.

Second-generation advanced high-strength steels, in contrast to first-generation steels, are highly alloyed (from 15% to 30% manganese) and have an austenitic structure at room temperature. Plastic deformation in these steels occurs in a twinning process [33]. Second-generation steels have an outstanding combination of strength and ductility due to their high manganese content, which gives austenite a stable structure at room temperature. The tensile strength and relative elongation of high-strength second-generation steels range from 900 MPa to 1600 MPa and from 45% to 70%, respectively [4]. A major disadvantage of these steels is their relatively high cost, caused by high manganese content and difficulties in welding, which limits the use of this group of high-strength steels.

Third-generation advanced high-strength steels are low- and medium-alloyed [31,34,35,36]. The microstructure of these steels possesses nano-sized structural components; the controlled gradient distribution of alloying elements between phases and the use of the transformation-induced plasticity effect provides a combination of high strength and plasticity [37,38,39,40,41,42].

Steels with a high carbon content are usually wear-resistant [43,44,45,46,47] and used as tool materials [48]. In special cases, carbon steels with a near-eutectoid composition, such as hypoeutectoid, hypereutectoid, and eutectoid steels with 0.8 wt.% C, can be treated to provide a high strength [49,50,51,52,53]. It is known that the isothermal treatment of eutectoid steel with subsequent plastic deformation provides a very high set of mechanical properties [51]. Such a treatment is usually carried out at temperatures from 500 °C to 550 °C, ensuring the minimum resistance of austenite to diffusion transformation. However, such processing can only be applied to a wire of a small cross-section, and thus the range of potential products that can be manufactured is significantly limited.

The motivation of this study was to discover a heat treatment method for eutectoid steel that could simultaneously increase its strength and ability to resist high-energy impacts.

## 2. Materials and Methods

The experiments were carried out using industrially produced 14 mm diameter rods made from unalloyed eutectoid steel containing 0.79 wt.% C. Table 1 shows the chemical composition of experimental steel, as provided by the supplier. The samples for tensile and impact mechanical tests were machined from these rods. 

Tensile tests were performed according to ISO 6892-1:2019 using proportional cylindrical samples with a working part diameter of 5 mm and lengths of 25 mm on a servo-hydraulic machine INSTRON 8801 (INSTRON, Norwood, MA, USA). Impact bending tests were performed according to ISO 148-1:2016 on an INSTRON IMP-460J (USA) pendulum machine using samples with dimensions of 7.5 mm × 10 mm × 55 mm, without a notch, and with a V-shaped notch. Due to the gradient microstructure of samples after heat treatment, samples without a notch were necessary for estimating the properties of the surface layer. 

The microstructure and fracture surfaces of the samples were studied using scanning electron microscopes, JEOL JSM-7000F and JEOL JSM-IT 300 (JEOL, Tokyo, Japan). Samples used for microstructural investigation were prepared by grinding with abrasive paper of different grain sizes, followed by polishing and etching in nital. After the impact tests, the fracture surfaces of the samples were examined using scanning electron microscopy without any additional processing.

The hardness of the samples was determined using a Vickers hardness tester TVP-5012 (TOCHPRIBOR, Ivanovo, Russia), load 98.1 N. To determine the microhardness, a MICROTECH HVA-1 (MICROTECH, Kharkiv, Ukraine) hardness tester was used at a load of 0.981 N. 

X-ray diffraction investigations were performed on a RIGAKU SMARTLAB SE X-ray diffractometer in Cu-Kα radiation (RIGAKU, Tokyo, Japan).

Samples were heated during heat treatment in a SUOL-0.44/12-M2-U42 (TULA-TERM, Tula, Russia) electrical resistance furnace. The temperature was measured using a chromel–alumel thermocouple. A temperature check was performed near the target values using a PP-63 (MICROPRIBOR, Lviv, Ukraine) portable potentiometer.

An unconventional heat treatment was applied to the samples, with the goal of maintaining the most finely dispersed ferrite–cementite microstructure by means of cooling from an austenitic single-phase area with a near-critical cooling rate. A near-critical cooling rate was provided via the controlled exposition of samples in the furnace at a temperature of 800 °C; thus, a different amount of cementite was dissolved in austenite. Therefore, the critical cooling rate of austenite was changed along with changing exposition. Because the actual cooling rate of samples was kept constant, it was assumed that there is an optimal exposition that would match the actual cooling rate and critical cooling rate at a given composition of austenite. On the one hand, heat treatment technologies only have a limited ability to refine the grain diameter of austenite; controlled plastic deformations with a combination of microalloying represent widespread possibilities for their formation [10]. On the other hand, the final secondary microstructure strongly depends on the state of austenite just before phase transformation. This state of austenite can be changed by different means. In our case, a certain chemical composition of austenite should be “fixed” by time-controlled holding. 

The temperature in the furnace was increased to the target value of 800 °C and maintained at a stable level for about 30 min to evenly heat the working space, the corundum bulb, and the lining of the furnace. After heating the furnace, the single sample was loaded into the furnace and subject to time-controlled holding, monitored using a stopwatch. After the end of exposure, the sample was quickly removed from the furnace with tongs and immersed in transformer oil at room temperature. This way, each sample was processed separately, ensuring the process was carried out at a stable speed. This ensured the stability of the heat treatment parameters, except for the time that the samples were kept in the furnace.

The time of exposure of each sample in the furnace varied from 3 to 9 min with an interval of 30 or 60 s. To clarify the optimal exposure time, the interval was reduced near the supposed optimal exposure.

## 3. Results

The results of impact tests of samples without notches are shown in Table 2.

**Figure 1:**
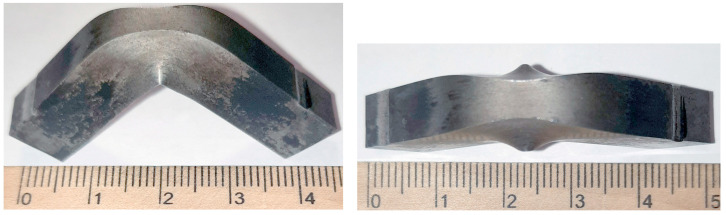
Impact sample after heat treatment with 7 min holding time and subsequent impact test.

**Figure 2:**
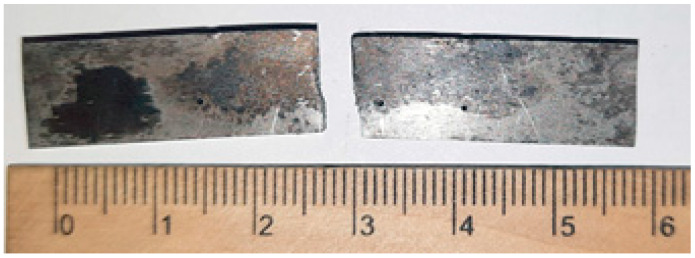
Impact sample after heat treatment with 8 min holding time and subsequent impact test.

The impact energy increased after the holding time exceeded 5 min. The sample after heat treatment with a 7 min holding time was not broken after impact with an energy value of 460 J (Figure 1). Figure 2 shows the broken tested sample, which was held in the furnace for 8 min. The cardinal difference between these two samples can be observed. A difference in holding time as small as one minute led to a drastic decrease in the impact energy of the steel samples.

The data in Table 2 show that after holding from 3.0 to 5.5 min, the impact toughness of steel is lower than that in the annealed state. Gradually increasing the exposure time leads to an increase in impact toughness up to a certain critical exposure time (7 min). Thus, a clear extreme dependence was established (with a minimum and a maximum) of the impact toughness on the exposure time of the eutectoid steel at a temperature of 800 °C before cooling.

Table 3 shows the results of tensile tests. The test results indicate the same extreme dependence of strength on exposure time as for impact samples.

The obtained results indicate that it is possible to strengthen eutectoid steels by controlled holding in the single-phase region and subsequent cooling in oil. The dwelling time is critical and must be precisely controlled to achieve the maximum strengthening effect.

**Figure 3:**
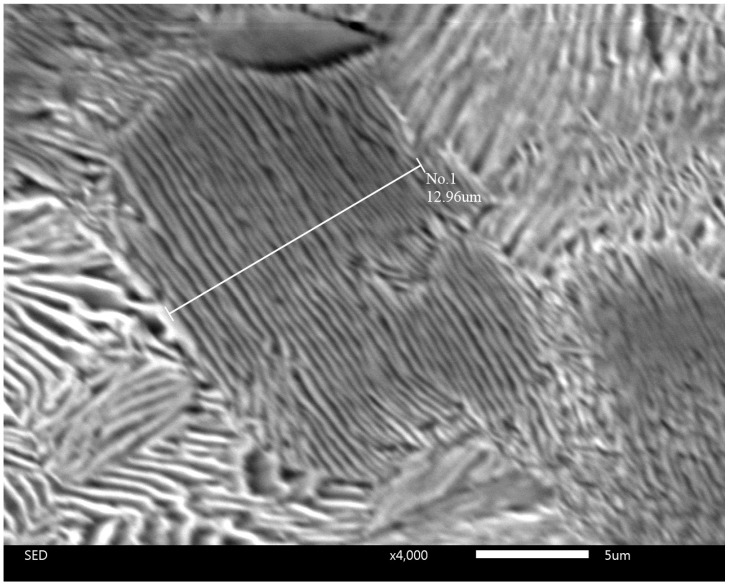
SEM image of eutectoid steel in the as-received state (annealing) with a pearlite colony 12.96 μm in size.

Figure 3 shows an SEM image of eutectoid steel in the as-received state (annealing) with a pearlite colony 12.96 μm in size (35 cementite plates, approximately 3 cementite plates per micrometer).

**Figure 4:**
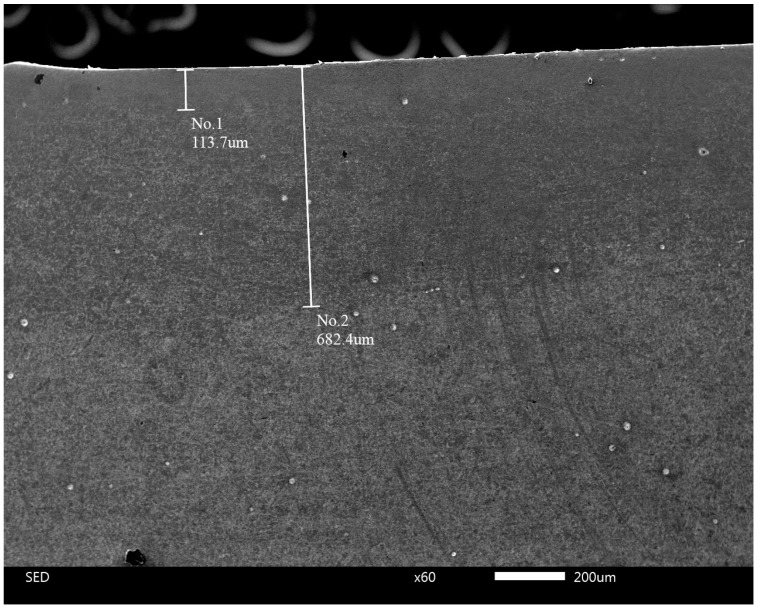
SEM image of a cross-section of the impact sample after heat treatment with a holding time of 6 min 30 s.

Figure 4 shows the SEM micrograph at a low magnification (×60) of a cross-section of the sample after heat treatment with a holding time of 6 min 30 s. Three different zones can be seen at different distances from the surface: to a depth up to 100 μm, from 100 μm to 680 μm, and deeper than 680 μm. Thus, the gradient microstructure is formed as a result of heat treatment with the controlled holding time.

**Figure 5:**
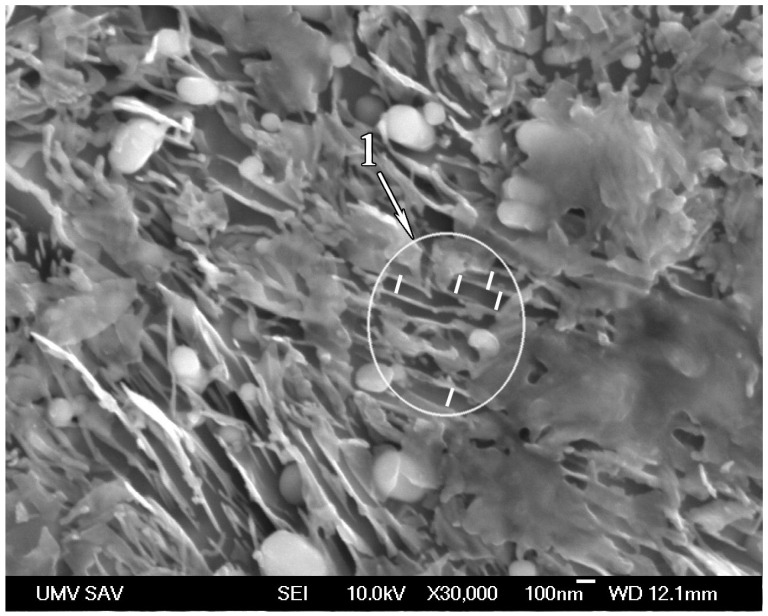
SEM micrograph of the under-surface region of heat-treated sample with a holding time of 6 min 30 s.

Figure 5 shows the microstructure of the same sample less than 100 μm from the surface. The structure contains undissolved carbides and cementite plates with an interlamellar space of approximately 100 nm (see Figure 5, zone 1). The presence of undissolved carbides indicates that carbon content in austenite just at the moment of cooling was less than 0.8 wt.%.

**Figure 6:**
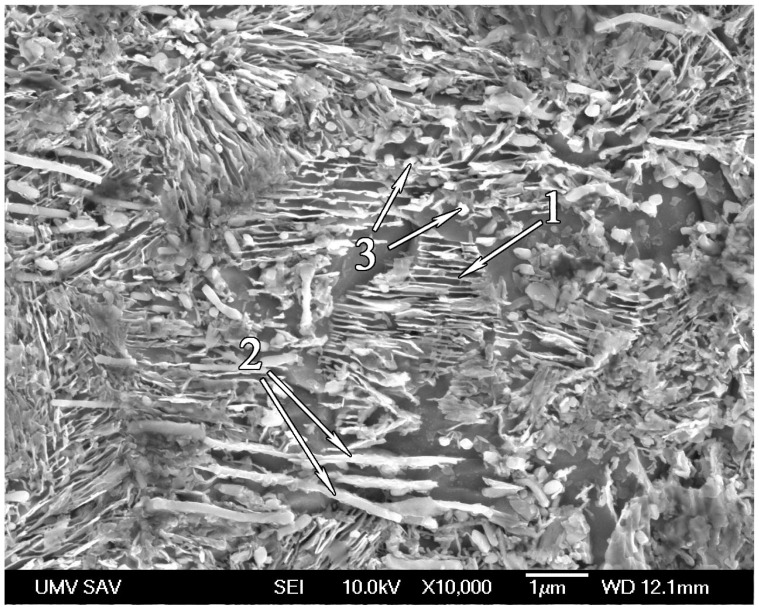
SEM micrograph of a heat-treated sample with a holding time of 6 min 30 s approximately 200 µm from surface: freshly formed thin lamellae of cementite (1), coarse undissolved cementite plates (2), and globular undissolved carbides (3).

Figure 6 demonstrates the microstructure of the same sample at approximately 200 µm from the surface. The distinctive microstructural constituents are freshly formed thin lamellae of cementite (1), coarse undissolved cementite plates (2) inherited from the past microstructural state, and globular undissolved carbides (3). Globular undissolved carbides (3) are likely the remains of dissolved cementite lamellae. 

**Figure 7:**
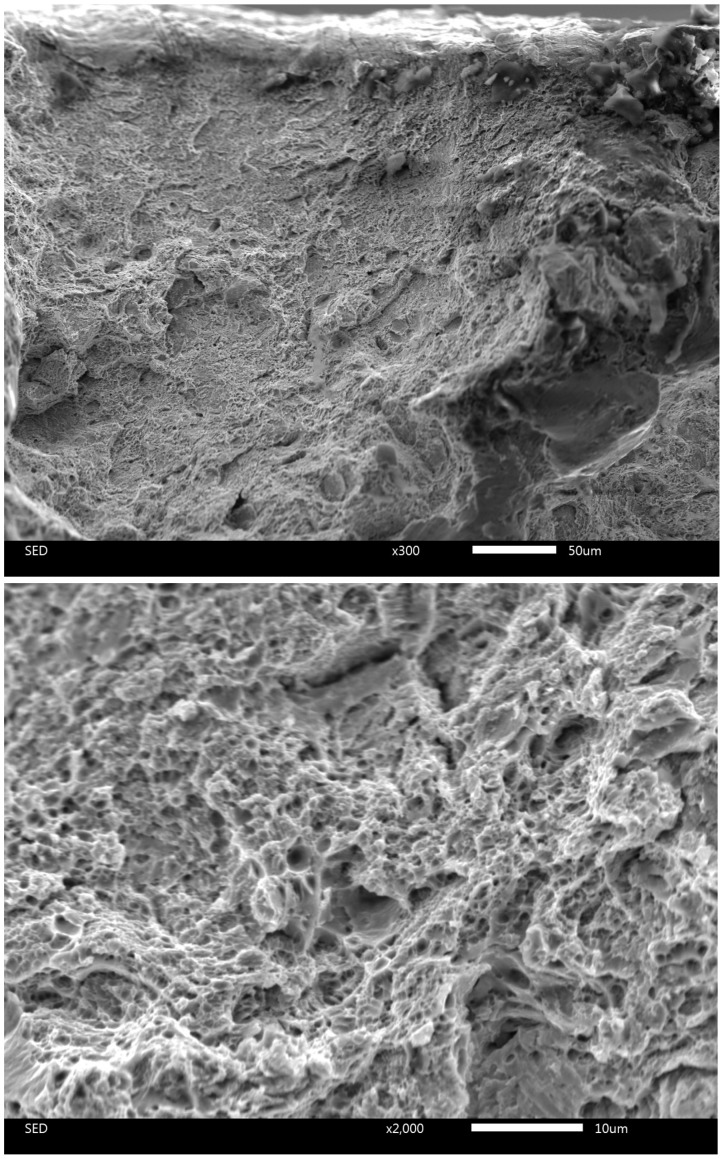
Fractogram at different magnifications of under-surface region of the sample after heat treatment with a 6 min 30 s holding time.

**Figure 8:**
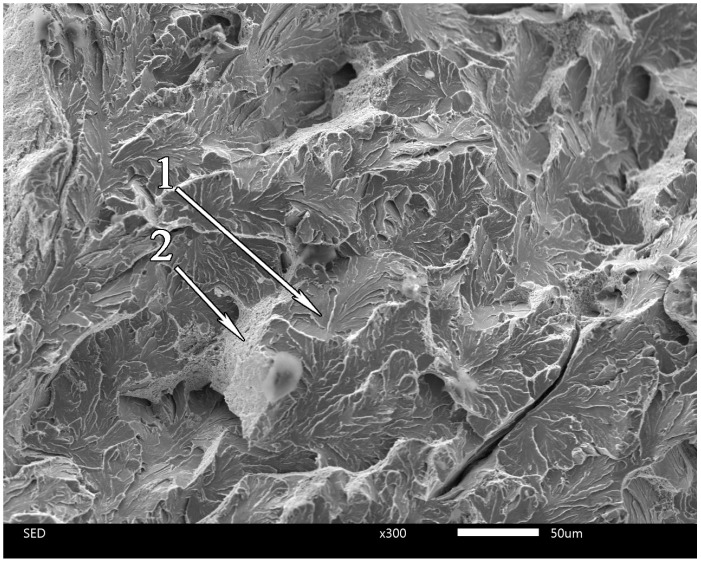
The fractogram of the central region of the sample after heat treatment with a 6 min 30 s holding time: quasi-brittle fracture (1) and ductile fracture (2).

The significant differences in microstructure at different depths from the surface should lead to different fracture modes of corresponding areas; Figure 7 and Figure 8 clearly demonstrate this difference. The fractogram in Figure 7 corresponds to the under-surface region of the sample after heat treatment with a holding time of 6 min 30 s. This is a typical ductile fracture. Images of the fracture surface shown in Figure 7 are identical to numerous examples of SEM images for ductile fracture ([54] Figure 10, [55] Figure 6, [56] Figure 25, etc.) Contrastingly, Figure 8 demonstrates a preliminary brittle fracture (1) of the central region of the sample after heat treatment with a 6 min 30 s holding time. The locations (1) of fracture on the surface, as shown in Figure 8, are identical to numerous examples of SEM images of brittle fracture ([54] Figure 11, [56] Figure 1a, etc.) Locations of ductile fracture (2) are also present, but their relative area is far less than those in the undersurface regions.

**Figure 9:**
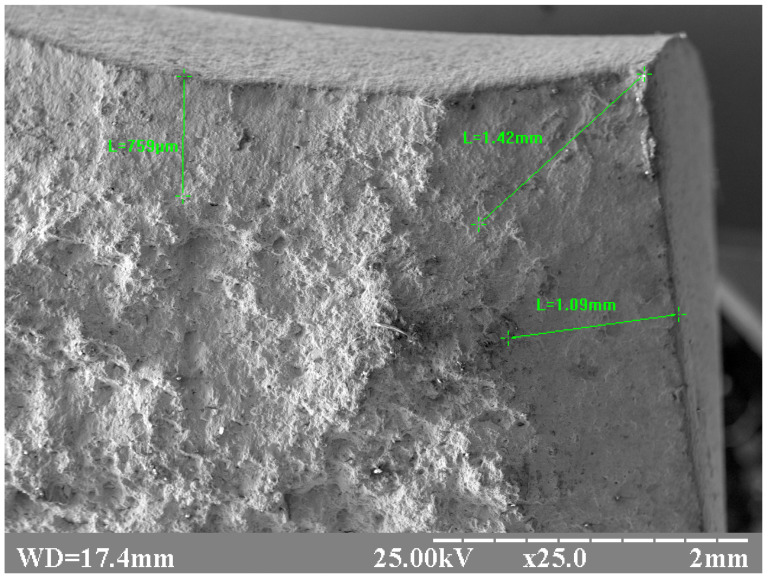
Low-magnification fractogram of the sample after heat treatment with a 6 min 30 s holding time.

A low-magnification SEM image of the same sample allows assessing the proportion of the plastic zone (Figure 9). The depth of the undersurface layer, with a different appearance of the fracture surface in comparison with the core surface, can be assessed as 0.7–1.0 mm. This observation correlates with the depth of the altered layer, which is shown on Figure 4.

**Figure 10:**
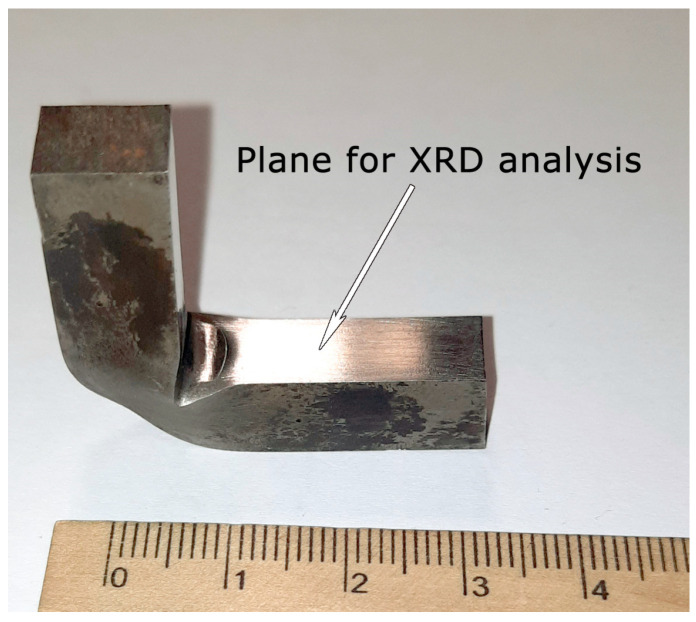
The location of the XRD investigation on the unbroken sample.

In order to characterize the material at the very surface, an XRD investigation was performed at the top of the unbroken sample, as shown in Figure 10. The location of the XRD investigation on the unbroken sample is shown by an arrow.

**Figure 11:**
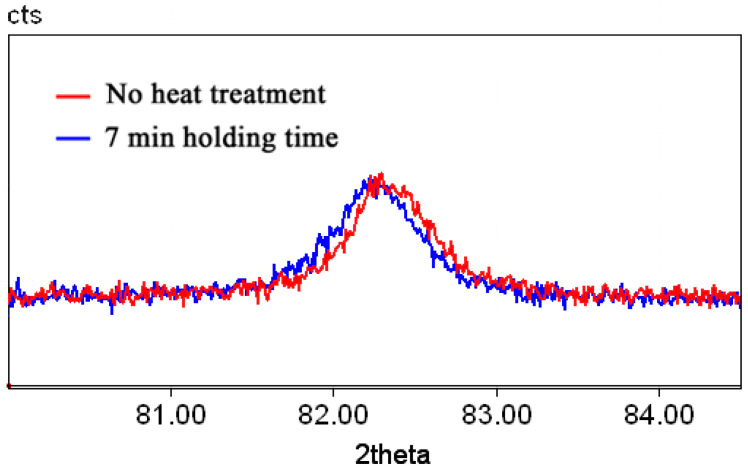
(211)α diffraction maximum of samples without heat treatment (red) and after heat treatment with a 7 min holding time (blue).

Figure 11 shows part of the diffractograms in the region of the (211)α diffraction maximum of the sample without heat treatment and after heat treatment with a 7 min holding time (undamaged sample, see Figure 1 and Figure 10). It was established that the (211)α diffraction maximum was shifted towards smaller values of 2Θ for the sample after a 7 min holding time. This shift indicates an increase in carbon content in the solid solution. 

## 4. Discussion

**Figure 12:**
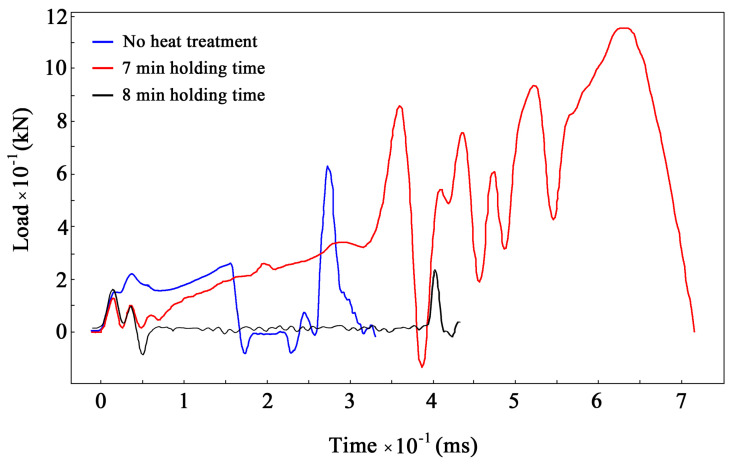
Load development during impact testing of samples without heat treatment (blue), after 7 min holding time (red), and after 8 min holding time (black).

Figure 12 shows a combined load–time diagram with three curves obtained after the impact tests of three samples: without heat treatment (blue), after 7 min holding time (red), and after 8 min holding time (black). Load values are detected with force censors that are mounted on the pendulum. Several distinctive observations can be made here.

The black curve clearly indicates load maximums at the very beginning and end of the fracture process. The first maximum corresponds to the fracture of the surface layer that is under tension. The second maximum corresponds to the fracture of the compressed layer. Between these two maximums, there is a long period of zero force. Evidently, this corresponds to crack propagation through the core of the sample. The blue curve has a similar but significantly shorter period of zero force. This observation leads to the conclusion that samples with initial notches (V, U, or T) do not allow for the estimation of the energy needed for the rupture of the surface layer. However, this same layer is the most heavily loaded in any construction during static or impact bending. Therefore, the impact tests of samples without notches should be carried out along with the testing of notched samples in order to more accurately estimate the fracture behavior of real parts.

The load curve for the unbroken sample has a steady growth area and region of significant load fluctuation. This effect can be attributed to internal friction in heavily deformed parts of the sample (see Figure 1). The stick–slip movement of internal layers presumably causes extremums of the load, and the friction force reaches its maximum at the moment when the motion starts.

Additionally, it is evident that the absolute load value for the unbroken sample is significantly higher than those of the other two, and the loading time is almost twice as long. As such, the value of energy that was adsorbed by the unbroken sample appears higher than the stored energy of the pendulum.

It should be noted that the V-notched sample after treatment with the same 7 min holding time has only 21 J of adsorbed impact energy. The drastic difference in impact energies for the unnotched and V-notched samples is observed in the clear difference between the corresponding fractograms (see Figure 7 and Figure 8). The surface area of the sample after treatment with a 6 min 30 s holding time demonstrates a predominantly ductile fracture (Figure 7). Contrastingly, the core of the sample is broken in predominantly brittle mode (Figure 8). Areas like those indicated by arrow 1 correspond to brittle fracture and occupy most of the image area (see Figure 8). Although, areas of ductile fracture (arrow 2) are also presented on the fractogram for the core of the sample.

According to [57], the hardness of the ferrite–cementite microstructural constituent depends on interlamellar spacing. The study was carried out for steel with a carbon content of 0.82% in the range of interlamellar spacing from 200 nm to 700 nm.

**Figure 13:**
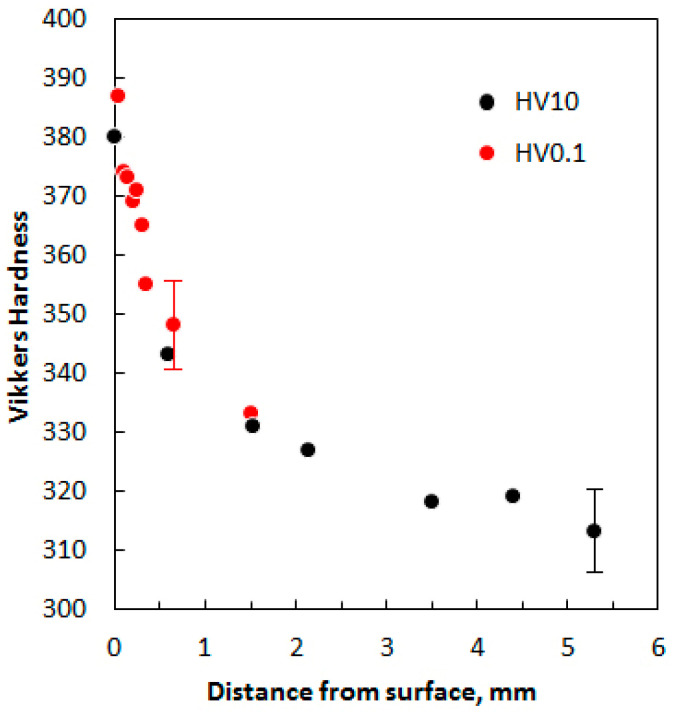
The correlation between the Vickers hardness of sample (6 min 30 s holding time) and distance from surface. The distance of 0 mm corresponds to the usual hardness measurement on the surface itself.

The interlamellar spacing in the ferrite–cementite mixture of the same steel after heat treatment with an optimal holding time is less than 100 nm (see Figure 5). For a comparison with existing data [57], the correlation between hardness and distance from the surface was determined using a cross-section of the impact sample after a 6 min 30 s holding time (see Table 2). Vickers hardness measurements were performed at different depths from the surface with indenter loads of 10 kg and 0.1 kg. The results are shown in Figure 13. It can be seen that the hardness increases from HV 315 in the core to almost HV 390 on the surface of the sample.

**Figure 14:**
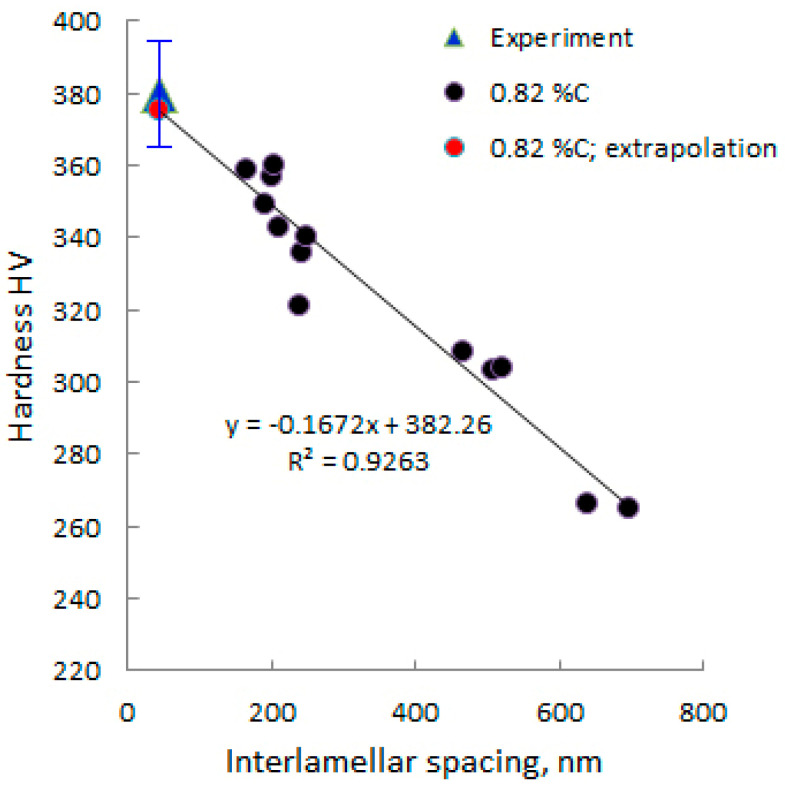
The correlation between Vickers hardness and interlamellar spacing in eutectoid steel [57].

It has been established that the actual obtained hardness coincides with the extrapolated hardness, according to data from [57] (Figure 14).

Thus, hardness can serve as an express indicator for determining whether or not a nanostructured state is achieved after thermal treatment. According to Figure 14, an interlamellar spacing of 100 nm corresponds to an approximate hardness of HV360. Therefore, in order to achieve a high resistance for an impact load, it is necessary to obtain the surface hardness of a sample or machine part at a level of HV360 or higher.

According to the nature of time-controlled thermal treatment, it is barely possible to obtain a nanostructured layer at a relatively large depth. Each point of the material at a certain depth has its own optimal holding time at high temperatures. It is evident that the surface layer is heated faster than deeper layers. Therefore, the optimal holding time for surface layer is minimal. This leads to the inevitable specific distribution of hardness with depth, which is demonstrated in Figure 12. This hardness distribution appears to be almost the same as that after some chemical–thermal treatment. But unlike chemical–thermal treatment, this method is much more time-efficient. 

The interlamellar spacing in pearlite was extensively studied and reviewed in [58]. It was shown that minimal interlamellar spacing (90 nm) corresponds to pearlite that is isothermally formed by lead patenting. As such, the proposed thermal treatment of eutectoid steel with a controlled holding time allows a microstructure to be obtained in the surface layer that is similar to that after lead patenting. Therefore, the use of the harmful and inconvenient technology of isothermal holding in lead baths can be avoided or significantly reduced. 

In Table 1, the impact energy increases with an increase in holding time when the holding time is larger than 4 min, demonstrating an improvement in toughness. However, as shown in Table 2, the relative elongation drastically decreases after a holding time of over 4 min, demonstrating a decrease in toughness. This phenomenon can be explained by taking into account different cross-sections of impact and tensile samples, as well as different modes of applying the stress during sample loading under bending and tension.

When the pearlite structure reaches a eutectoid temperature, α-γ phase transition takes place as the first stage of transformation. The second stage is dissolving cementite in austenite. The exact time when a certain point at a certain depth reaches the threshold temperature depends on heat flow into the sample from surrounding hot media and heat dissipation from the surface inside the sample. The higher the temperature of the sample core, the less intensive the heat flow from surface to the center and the shorter the time needed for the surface to reach eutectoid temperature. For a sample with a smaller cross-section, the core heats up faster. Hence, the surface of a sample with a smaller cross-section reaches eutectoid temperature faster. These considerations explain why the optimal time for a tensile sample with a 5 mm diameter (4 min, see Table 2) exceeds that time needed for an impact sample with a 7.5 × 10 mm^2^ cross-section (7 min, see Table 1).

The common feature between impact and tensile samples is that both of them rapidly lose plasticity when the holding time exceeds the optimal time. This is explained by the appearance of brittle high-carbon martensite after cooling. However, the significant difference is that the impact sample has a high plasticity after treatment under the optimal time, while the tensile sample has a decreased plasticity when treated at the highest strength. This can be explained by the gradient microstructure of samples after thermal treatment (see Figure 4 and Figure 12). The external zone of samples is harder, and thus stronger, than the material in the core. During impact, the bending of a sample takes place, with the most stress and deformation located in the external zone. Therefore, the core is significantly less stressed and deformed before the crack appears at the surface, meaning that the impact sample can affect the continuity of the core material, while the external zone bears high stress and deformation. Tensile samples are normally loaded; therefore, stresses are developed uniformly across the cross-section. The core zone has less strength and begins to plastically deform earlier than in the external zone. Even small cracks inside the sample in the core would lead to the concentration of stress and brittle rupture of the remaining material. It is known that under certain conditions, even materials with high plasticity possess brittle fracture if cracks exist in the sample or a structural element [59]. Nevertheless, this question is very important and further research is needed to establish the peculiarities of fracture of carbon steel with gradient microstructure under normal load. Additionally, it is necessary to make an effort to optimize the holding time depending on the dimensions of the element that is subjected to heat treatment.

The important question is as follows: what type of metal matrix are cementite lamellae located in? The RXD investigation of the surface of the unbroken sample after a 7 min holding time shows a shift of a (211)α diffraction maximum towards smaller values of 2Θ (see Figure 9). The calculation according to the method of [60] shows that the solid solution of the heat-treated sample after a 7 min holding time contains 0.12 wt.% of carbon. In fact, the nanostructured composite is formed from thin cementite lamellas and low-carbon martensite. Thus, the similarity is shown (or “established” or “determined”) between the nanostructured state of carbon steel after heat treatment with a controlled holding time and the structure obtained in eutectoid steels after cold plastic deformation of several hundred percent [49].

Currently, carbon eutectoid steel is known to be the strongest metallic bulk material [51] when interlamellar spacing is decreased to few nanometers [49,50]. In such conditions, the steel possess about 30% of its theoretical strength, although a very high strain is needed to achieve such properties. In [49], a 6.02 true strain is reported to achieve a 6.32 GPa tensile strength in a pearlitic steel wire of hypereutectoid composition (0.98%C). In [50], the authors reported a 7 GPa tensile strength after drawing a patented eutectoid steel to a 6.52 true strain. The wire diameter after the last deformation step was 0.02 mm. These results indicate that carbon eutectoid steel possess high potential as a high-strength material. Our research demonstrates a method for creating nanostructures in bulk parts made from pearlitic steel without the complicated operations of patenting and cold drawing. Although an interlamellar spacing close to a few nanometers is not achieved in our study, in comparison with other results reported for bulk specimens after heat treatment, a significant decrease in this parameter was shown [57] (see Figure 14). Further research is needed to clarify the peculiarities of fracture mechanisms of pearlitic steel with a gradient microstructure in different modes of load application.

## 5. Conclusions

Unconventional heat treatment comprising controlled heating at 800 °C and cooling in oil was applied to carbon eutectoid steel. The main findings can be summarized as follows.

- There is a certain optimal holding time at a high temperature that should be precisely maintained, with subsequent sample cooling in oil. This controlled holding time provides an incomplete dissolution of cementite and therefore a precise match of the critical cooling rate of austenite and actual cooling rate during cooling in oil. As a result, a very fine pearlitic nanostructure appears at depth of approximately 100 μm from the surface, with an interlamellar spacing of approximately 100 nm and a hardness of HV 380–390. This nanostructure provides a significant increase in resistance to impact load. After a heat treatment with the optimal holding time, the impact sample with a cross-section of 7.5 × 10 mm^2^ was not broken after impact with an energy value of 460 J.
- The optimal holding time strongly depends on the sample cross-section. It was established that the optimal holding time is 7 min for the impact sample, with a cross-section of 7.5 × 10 mm^2^ and 4 min for the tensile sample, which is 5 mm in diameter. Excessive holding at a high temperature before cooling leads to appearance of high-carbon martensite and a drastic decrease in impact toughness and strength. Therefore, for each given section of a machine part, special preliminary experiments are necessary to establish the optimal exposure time at a high temperature.
- The XRD investigation showed that the solid solution of the heat-treated impact sample after a 7 min holding time contains 0.12 wt.% of carbon. It means that a nanostructured composite is formed that comprises thin cementite lamellas located in low-carbon martensite.
- Further research is needed to better understand the fracture mechanism of the obtained gradient microstructure of high-carbon steel under a tensile load. Additionally, it is necessary to establish the range of dimensions of parts that are suitable for heat treatment with controlled holding at a high temperature. This research will promote a better understanding of how the changes in shape, sample thickness, and surface quality would affect the properties of high-carbon steel after time-controlled thermal treatment.
- The obtained results can be used for the production of machine parts and structural elements for which high resistance to impact loads is critically important.

## Figures and Tables

**Table 1:** Chemical composition of industrially produced eutectoid steel, wt.%.

C	Si	Mn	Cr	Ni	Cu	P	S
0.79	0.23	0.21	0.19	0.17	0.22	0.011	0.004

**Table 2:** The impact testing results of carbon eutectoid steel after heat treatment with controlled holding. Samples without notches.

Holding Time, min-s	Impact Energy, J
No heat treatment	218
No heat treatment	41 ^1^
3-00	100
4-00	109
4-30	153
5-00	141
5-30	281
6-00	237
6-30	Sample was half-broken. Gauge was stopped at the bottom low position
6-30	8 ^1^
6-45	25 ^1^
7-00	Sample was not broken. Gauge was stopped by the sample
7-00	21 ^1^
8-00	18
9-00	10

^1^ V-notched sample.

**Table 3:** The tensile testing results of carbon eutectoid steel after heat treatment with controlled holding. Load speed: 5 mm/min.

Holding Time, min-s	Yield Strength, MPa	Tensile Strength, MPa	Relative Elongation, %	Relative Cross-Section Reduction, %
No heat treatment	903	1043	6.8	19.0
1-30	628	1045	11.6	20.3
2-00	675	1033	8.0	21.5
2-30	569	1029	10.8	21.5
3-00	543	1018	8.0	25.1
3-30	513	1091	8.0	21.9
4-00	1187	1295	1.5	-
4-30	1000	1000	-	-
5-00	873	873	-	-
6-00 *	341	341	-	-
8-00	658	658	-	-

* Sample was broken in brittle mode under a load of 17,069 N over the head cross-section with area of 50 mm^2^.

## Data Availability

The original contributions presented in the study are included in the article, further inquiries can be directed to the corresponding authors.
